# Clinical efficacy and mechanism of the combination of autologous platelet-rich gel and recombinant human acidic fibroblast growth factor in the management of refractory diabetic foot

**DOI:** 10.3389/fendo.2024.1374507

**Published:** 2024-10-30

**Authors:** Xia Sheng, Ling Hu, Ting Li, Yi Zou, Hai-Yan Fu, Guo-Ping Xiong, Yan Zhu, Bo Deng, Lei-Lei Xiong, Xiao-Ling Yin

**Affiliations:** Department of Metabolism and Endocrinology, The Third Affiliated Hospital of Nanchang University First Hospital of Nanchang, Jiangxi Provincial Key Laboratory of Metabolism and Endocrinology, Nanchang, China

**Keywords:** diabetic foot, oxidative stress, pigment epithelium-derived factor, platelet-rich gel, vascular endothelial growth factor

## Abstract

**Objective:**

This study aims to explore the influence of combining autologous platelet-rich gel (APG) with continuous vacuum-sealed drainage (CVSD) and the exogenous recombinant human acidic fibroblast growth factor (rh-aFGF) on the healing processes of diabetic foot ulcers (DFU). The primary objective is to elucidate the complex molecular mechanisms associated with DFU, providing innovative perspectives for its treatment.

**Methods:**

Ninety patients diagnosed with DFU were randomly allocated into three distinct groups. Group A underwent CVSD following wound cleansing to facilitate healing. In Group B, in addition to conventional treatment, negative pressure wound therapy was applied, and rh-aFGF was introduced into normal saline for lavage, building upon the procedures of Group A. Group C received APG along with the interventions applied in Group B. The clinical efficacy of each group was systematically observed and analyzed. Additionally, changes in plasma oxidative stress, inflammatory markers, vascular endothelial growth factor (VEGF), and pigment epithelium-derived factor (PEDF) were assessed both before treatment and 14 days post-treatment.

**Results:**

Following treatment, all groups exhibited commendable clinical efficacy. Group C demonstrated a superior wound healing rate, reduced frequency of dressing changes, and shorter wound healing duration (P< 0.05). Compared to baseline measurements, the levels of superoxide dismutase and PEDF increased, while malondialdehyde, VEGF, interleukin-6, interleukin-8, and monocyte chemotactic factor MCP-1 decreased in the wound tissue across all groups. Notably, Group C showed the most significant improvement in clinical efficacy and fortification of molecular mechanisms against oxidative stress (all P< 0.05).

**Conclusions:**

The integrative therapeutic approach combining APG with CVSD and rh-aFGF demonstrates notable efficacy in advancing wound healing. This effectiveness is evident through the reduced frequency of dressing changes and alleviation of wound-related pain. Additionally, the treatment regimen improves the cure rate for challenging, refractory wounds. These favorable outcomes can be attributed to the reduction of oxidative stress levels, attenuation of the local inflammatory response, and the enhancement of the balance between PEDF and VEGF.

## 1 Introduction

Diabetic foot ulcers (DFU) represent a severe and enduring complication of diabetes and have emerged as a predominant cause of disability and mortality among chronic diseases. This condition places a substantial burden on both patients and society at large. The complex pathogenesis of DFUs involves elevated levels of oxidative stress, glycosylation, inflammation, and an imbalanced distribution of growth factors, all of which significantly contribute to the challenging healing process of DFUs. Reducing excessive reactive oxygen species, promoting an anti-inflammatory response, and maintaining a stable growth factor equilibrium are pivotal for the effective treatment and healing of DFUs (1). Despite ongoing clinical efforts, the outcomes of DFU treatments remain less than satisfactory. Current investigations are increasingly focused on assessing the efficacy of platelet-rich plasma (PRP) combined with continuous negative pressure sealing drainage (CVSD) for DFU treatment; however, the reported results remain inconsistent (2). Negative pressure wound therapy (NPWT) is a technique that promotes wound healing by applying negative pressure to the wound site. Platelet-rich plasma (PRP) therapy, on the other hand, involves extracting plasma rich in platelets from the patient’s own blood and reinjecting it into the wound or damaged tissue. PRP contains a high concentration of growth factors, which stimulate cell regeneration and repair, speeding up the healing process. The primary difference between NPWT and PRP lies in their treatment methods and mechanisms of action. NPWT promotes wound healing through a physical approach (negative pressure), while PRP facilitates tissue repair through biological substances (growth factors). Autologous platelet gel (APG) is a gel-like substance made from the patient’s own blood, offering several advantages over traditional PRP: (1) Gel-like consistency: The gel form of APG allows it to remain localized in the treatment area after injection, minimizing platelet loss and enhancing therapeutic efficacy. (2) Sustained release of factors: APG can continuously release growth factors and other bioactive substances over time, providing a longer-lasting treatment effect. (3) Stability: The preparation process of APG enables better control of platelet concentration and activity, ensuring consistent and effective treatment. (4) Reduced cell damage: APG is prepared using a gentle process that minimizes damage to the platelets, preserving their integrity and function. (5) Safety: Since APG is derived entirely from the patient’s own blood, it eliminates the risk of immune rejection associated with donor materials, ensuring higher safety. (6) Personalized treatment: APG can be tailored to the patient’s specific needs by adjusting preparation parameters, allowing for personalized treatment plans. Consequently, in this study, we aim to enhance the understanding of the effectiveness of APG in conjunction with CVSD in patients with DFU. The objective is to explore additional systematic and feasible treatment options.

Recombinant human acidic fibroblast growth factor (rh-aFGF) enhances both the proliferation and differentiation of fibroblasts and vascular endothelial cells within granulation tissue, thereby expediting granulation tissue growth. This biological mechanism is pivotal in promoting the healing process of DFU. However, it is important to recognize that the underlying tissue pathophysiology imbalance in DFU cannot be effectively rectified through the administration of a singular growth factor or relying on a singular treatment modality (3).

PRP represents a concentrated platelet formulation derived from the centrifugation and separation of whole blood. It contains various cytokines and growth factors known to facilitate wound healing, making it a widely employed therapeutic intervention for acute conditions such as arthritis and burns. Notably, over the past few years, there has been an escalating research interest, both domestically and internationally, in exploring the application of PRP for the treatment of chronic DFUs. However, further animal studies and clinical trials are needed to comprehensively assess its efficacy and potential adverse reactions (4, 5). NPWT has emerged as a significant adjunctive technique in promoting wound healing. Its benefits include the creation of a conducive, moist environment for rapid epithelization, as well as the facilitation of timely drainage to reduce tissue edema and minimize the risk of wound infection. Recognizing the limitations of single treatments in clinical practice, this study endeavors to systematically explore comprehensive treatment approaches.

Building upon the examination of traditional treatment regimens, this study investigates the modifications and clinical efficacy resulting from the application of exogenous rh-aFGF alone or in combination with NPWT and APG comprehensive therapy for DFU. Malondialdehyde (MDA) and superoxide dismutase (SOD) serve as indicators of oxidative stress, while interleukin-6 (IL-6), interleukin-8 (IL-8), and monocyte chemotactic factor 1 (MCP-1) are used as markers of inflammation. Vascular endothelial growth factor (VEGF) and pigmented epithelium-derived factor (PEDF) are assessed as growth factors. The primary objective is to further elucidate the clinical efficacy and molecular mechanisms underlying the enhancement of DFUs. Ultimately, we aim to provide a systematic and comprehensive treatment plan for the management of diabetic foot wounds, offering novel insights for future research endeavors.

## 2 Materials and methods

### 2.1 Study materials

Among the 456 patients with DFU who were hospitalized in the Department of Endocrinology and Metabolism of our hospital from June 2022 to December 2023, a total of 90 patients were voluntarily enrolled in this study after signing informed consent forms. Support for this study was provided by the clinical departments, and the medical Ethics Committee of our hospital approved it. Inclusion criteria were as follows: 1) Patients meeting the 1999 WHO diagnostic criteria for diabetes; 2) Patients meeting Wagner grade 2–4 of DFU; 3) Patients aged 18–85 years old. Exclusion criteria included: (1) Pregnant or lactating women; (2) Patients allergic to recombinant fibroblast components; (3) Patients with diabetic ketoacidosis or nonketotic hyperosmolar coma; (4) Patients with ankle-brachial index (ABI) less than 0.5 and/or combined with acute osteomyelitis; (5) Patients with local ulcers accompanied by tumors such as skin cancer or breast cancer; (6) Patients with ulcer area larger than 40 cm²; (7) Patients with platelet count below< 100×10^9L; (8) Patients with severe anemia defined as hemoglobin levels below< 60 g/L; (9) Patients with abnormal liver function (Glutamyl transferase > 3x upper limit of normal (ULN), total bilirubin > 3x ULN) and glutamyl transpeptidase > 3x ULN), and renal abnormalities (Creatinine > 200 μmol/L); (10) Patients diagnosed with malignant tumors; (11) Patients with heart failure classified as NYHA class III–IV; (12) Patients suffering from mental disorders; (13) Abnormal coagulation function.

### 2.2 Experimental grouping

Ninety patients diagnosed with diabetic foot ulcers (DFU) were randomly assigned to three distinct groups. Initially, relevant confounding factors were excluded based on specific criteria. Subsequently, patients were stratified and randomly assigned according to the severity of their DFU, classified by Wagner grades 2-4. A semi-randomization approach was employed, where patients were first sorted by their admission dates and then grouped. This method ensured that confounding variables were evenly distributed across the groups, minimizing their impact on the results. Additionally, during data analysis, a multivariate regression model was used to control for multiple confounding variables. Conventional treatment was administered to all participants in this study, and the enrollment scheme was determined based on patient preferences and wound depth. In Group A (30 cases), the wound infection was effectively managed through conventional treatment, underwent CVSD following wound cleansing to facilitate wound healing. Group B (n = 30): In addition to conventional treatment, negative pressure wound therapy was applied, and the wound was continuously irrigated with a mixture of rh-aFGF and 250 ml normal saline to control infection. Group C (30 cases): Expanding upon the interventions in Group B, APG was administered to fill the wound sinus tract. Conventional treatment included a bacterial culture of local ulcer secretions or deep necrotic tissue, followed by drug sensitivity testing. Antibiotic therapy was selected based on etiology and drug sensitivity results. Standard ulcer debridement and dressing changes were implemented to eliminate infected secretions and necrotic tissue, control infection, enhance microcirculation, and reduce blood glucose levels, blood pressure, and lipid levels.

### 2.3 Experimental materials

(1) The procedures involved in the production and application of APG for wound infection control are outlined as follows: Following the reduction of exudate, APG treatment was administered to diabetic foot wounds, and a local oil bandage was applied to cover the treated area. The preparation of APG encompassed the following steps: Peripheral venous blood, amounting to 10 times the volume of the ulcer surface, was drawn. Platelet-rich plasma was acquired through two rounds of centrifugation. Subsequently, APG was combined with thrombin and 10% calcium gluconate in a ratio of 10 mL:20000 IU:1 mL. The resulting APG gel was either sprayed onto the ulcer surface using a syringe or injected into deep sinuses. Finally, Vaseline gauze was applied to cover the treated area. Dressings were maintained for 3 days initially, followed by replacement every 3 days thereafter for the ongoing evaluation of wound condition. Wound tissue samples were collected before the commencement of treatment, while granulation tissue samples were obtained on day 14 post-treatment.

(2) Antibodies used in the study: PEDF (bs-20784R, Bioss, 1/200), VEGF (bs-1313R, Bioss, 1/200), IL-6 (DF6087, Affinity, 1/100), IL-8 (DF6998, Affinity, 1/100), MCP1 (DF7577, Affinity, 1/100).

(3) MDA kit (E-EL-0060c, Gene Creat); SOD Kit (A001-3-2, Nanjing Jiancheng Bioengineering Institute).

(4) Recombinant human acidic fibroblast growth factor (rh-aFGF): Shanghai Tenry Pharmaceutical Co., Ltd.

(5) Negative pressure wound protection drainage material: Wuhan VSD Medical Science& Technology Co., Ltd.

### 2.4 Statistical analysis

Continuous variables were represented as the mean ± standard deviation (SD) for normally distributed data. Categorical variables were represented as numbers and percentages (%). Group comparisons were examined through one-way ANOVA, if the result of the one-way ANOVA obtained statistical significance, a *post-hoc* Wilcoxon Signed Rank test was performed to compare every two conditions. To control for multiple comparisons, the significance level for each individual test was adjusted by dividing the overall significance level by the number of comparisons conducted. Results of before and after treatment comparisons were examined through an independent sample t-test. The significance level for the tests was set at α=0.05.

## 3 Study results

### 3.1 Comparison of clinical efficacy among different treatment groups

The study included 60 male and 30 female patients, with a male-to-female ratio of 2:1 and an average age of 68.75 ± 8.93 years. The severity of diabetic foot ulcers was classified as follows: 9 cases of Wagner grade 2 (3 cases in each group), 75 cases of Wagner grade 3, and 6 cases of Wagner grade 4 (2 cases in each group). There were no occurrences of acute heart failure, severe allergic reactions, major amputations, or deaths. Various parameters, including age, duration of disease, pre-treatment Visual Analogue Score (VAS), frequency of dressing changes, post-treatment VAS, hospitalization duration, and wound healing time for each treatment group were observed and analyzed. The results, as depicted in **Table 1**, indicated no statistically significant differences in age, duration of disease, and pre-treatment VAS among the three groups (P > 0.05). However, significant differences were noted in the number of dressing changes, post-treatment VAS scores, hospitalization duration, and wound healing time between the groups (P< 0.05). Compared to Group A, both Groups B and C demonstrated shorter dressing change frequencies, reduced hospitalization durations, and faster wound healing times. Additionally, their post-treatment VAS scores were lower, with Group C exhibiting superior outcomes.

**Table 1:** Comparison of clinical efficacy in different treatment group(n=30, $\bar{X}$ ±S).

Group	Years	Course	Frequency of dressing change	VAS		LOS	Healing time
				Before treatment	Post treatment		
A	64.4±13.15	14.83±7.86	45.29±21.24	6.16±2.28	3.36±2.28	35.58±14.68	56.88±10.64
B	65.68±12.72	15.60±5.98	30.58±11.57*	6.12±2.43	2.36±1.54*	27.56±10.57*	35.64±10.78*
C	64.86±10.08	14.96±5.65	21.36±9.28*#	6.76±2.63	1.89±1.56*#	23.34±6.38*#	29.72±9.23*#

*Compared with group A, the difference was statistically significant, P<0.05; ^#^Compared with group B, the difference was statistically significant, P<0.05.

### 3.2 Change of wound area in different treatment groups

**Figure 1:**
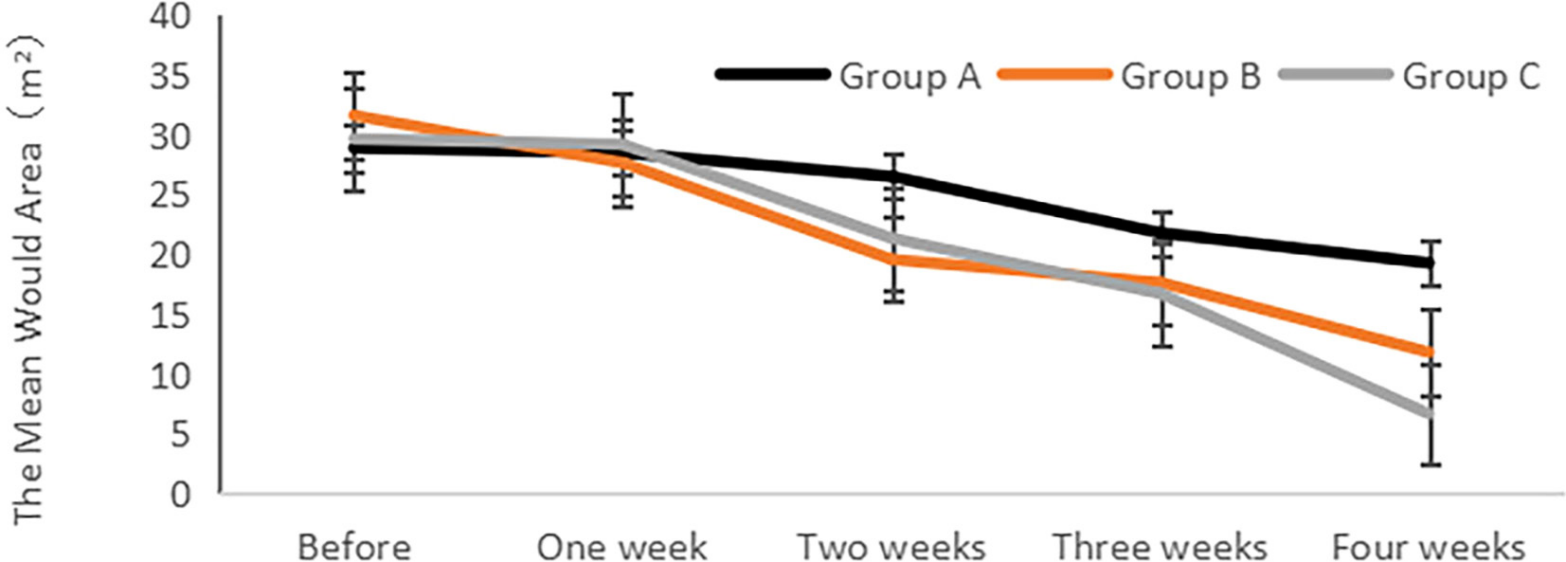
Change in wound area (m2) in different treatment groups.

The analysis of the wound area (length × width × sinus tract/stealth) for each group was conducted before and after treatment, as illustrated in **Figure 1**. The findings revealed no significant differences in the wound area among the groups before treatment (P > 0.05). However, after treatment, a notable improvement in the wound area was observed for each group in comparison to their respective pre-treatment values, reaching statistical significance (P< 0.05). Additionally, a gradual reduction in the wound area was observed post-treatment, with Group C demonstrating the smallest wound area and the shortest healing time.

### 3.3 Changes in oxidative stress markers—SOD and MDA before and after treatment in various treatment groups

**Figure 2:**
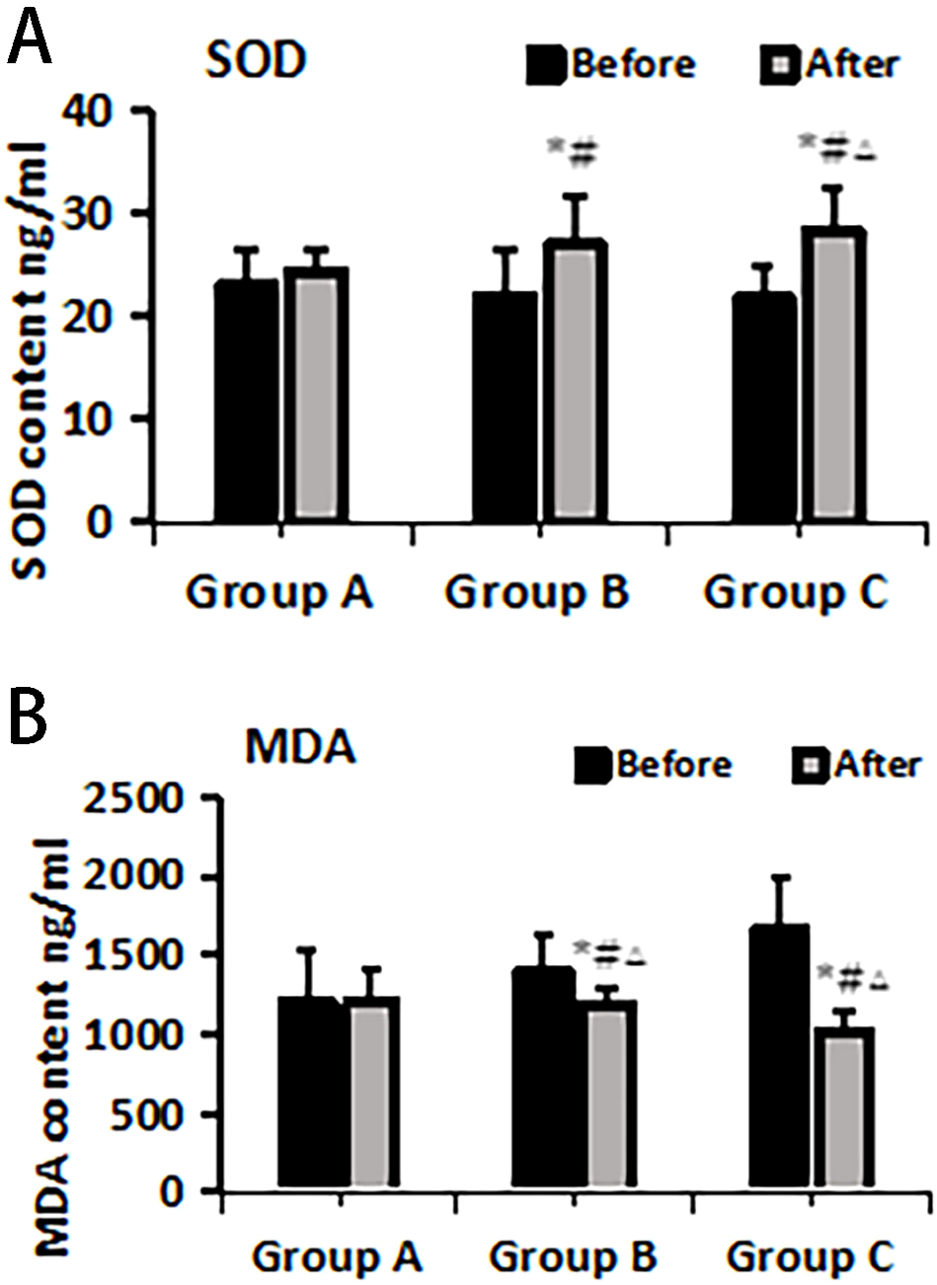
SOD **(A)** and MDA **(B)** changes pre- and post-treatment in different treatment groups. *Statistically significant difference was observed compared with the pre-treatment values, P< 0.05; #Statistically significant difference was observed compared with Group A, P< 0.05; △Statistically significant difference was observed compared with Group B, P< 0.05.

The ELISA method was employed to assess the levels of oxidative stress markers, specifically SOD and MDA, in plasma before and after treatment within each group. The results indicated no significant differences in SOD and MDA levels among the groups before treatment (P > 0.05). Following treatment, there was a substantial increase in SOD levels across all groups, with Group C exhibiting the most pronounced upregulation compared to Groups A and B, and the differences were statistically significant (P< 0.05). Additionally, MDA levels significantly decreased after treatment in all groups compared to pre-treatment values, with Group C demonstrating the most notable decrease compared to both Groups A and B and the differences were statistically significant (P< 0.05) (**Figure 2**).

### 3.4 Changes in VEGF and PEDF before and after treatment in different treatment groups

**Figure 3:**
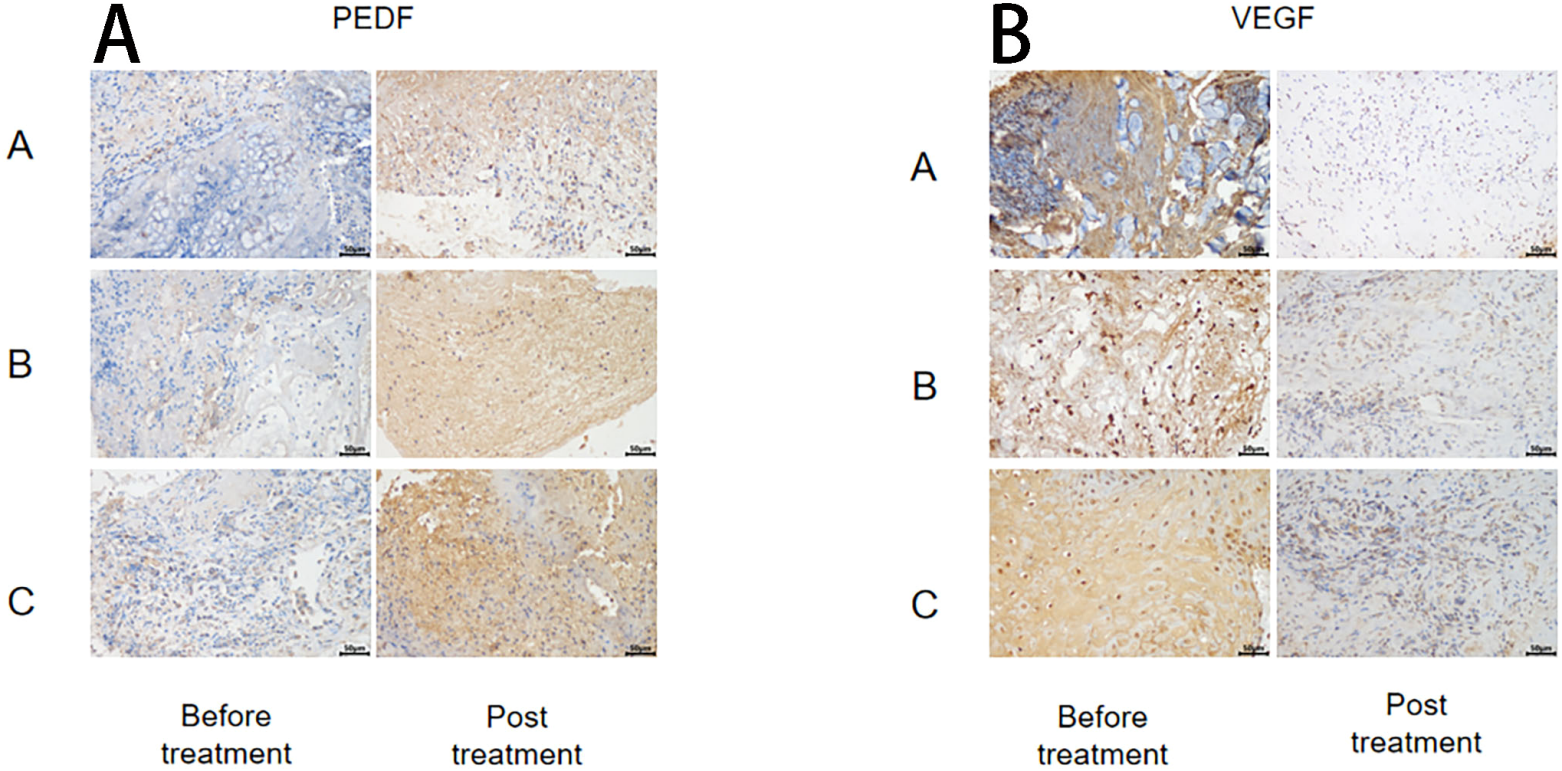
Immunohistochemical plots of PEDF **(A)** and VEGF **(B)**.

The immunohistochemical method was used to assess the changes in anti-vascular PEDF and VEGF levels in wound tissue before and after treatment within each group (**Figure 3**). The results demonstrated no significant differences in SOD and MDA levels before treatment among all groups (P > 0.05). After treatment, the PEDF levels significantly increased in each group compared to before treatment. Group C exhibited the most prominent elevation in PEDF levels, and this difference was statistically significant when compared with both Group A and Group B (P< 0.05).

**Figure 4:**
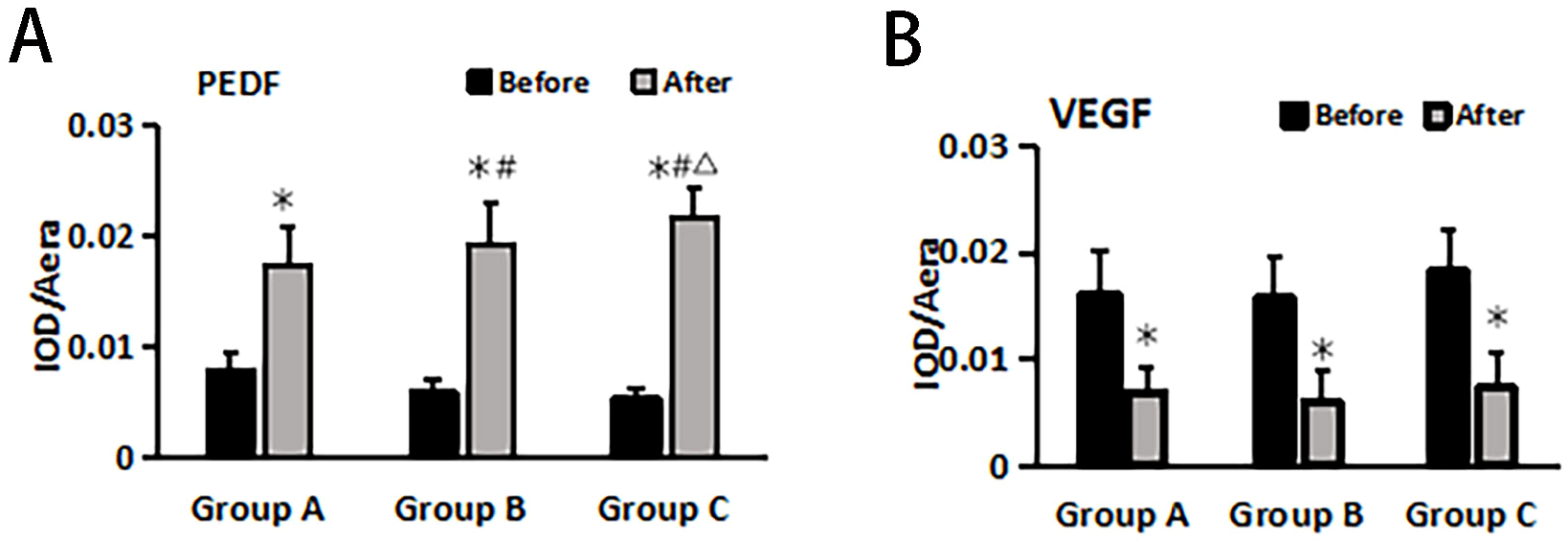
PEDF **(A)** and VEGF **(B)** changes before and after treatment in different treatment groups. *Statistically significant difference was observed compared with the pre-treatment values, P< 0.05; #Statistically significant difference was observed compared with Group A, P< 0.05; △Statistically significant difference was observed compared with Group B, P< 0.05.

Following treatment, VEGF levels significantly decreased within each group compared to pre-treatment values (P< 0.05), but there were no notable differences between the groups themselves (P > 0.05) (**Figure 4**).

### 3.5 Changes in inflammatory markers IL-6, IL-8, and MCP-1 after treatment in different treatment groups

**Figure 5:**
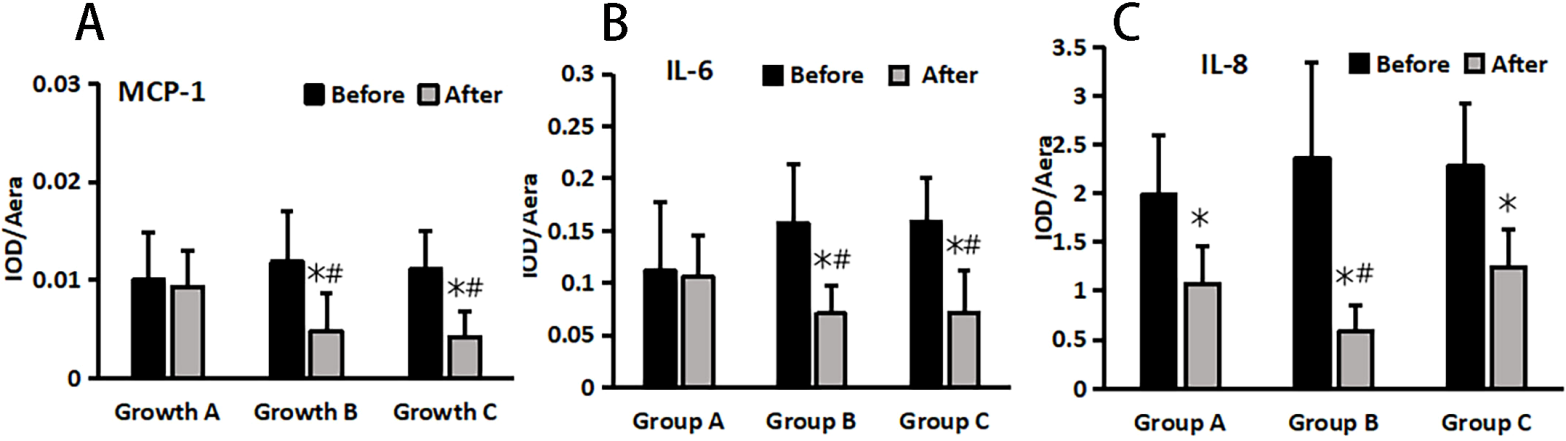
MCP-1 **(A)**, IL-6 **(B)**, and IL-8 **(C)** changes pre and post treatment in different treatment groups. *Statistically significant difference was observed compared with the pre-treatment values, P< 0.05; #Statistically significant difference was observed compared with Group A, P< 0.05.

**Figure 6:**
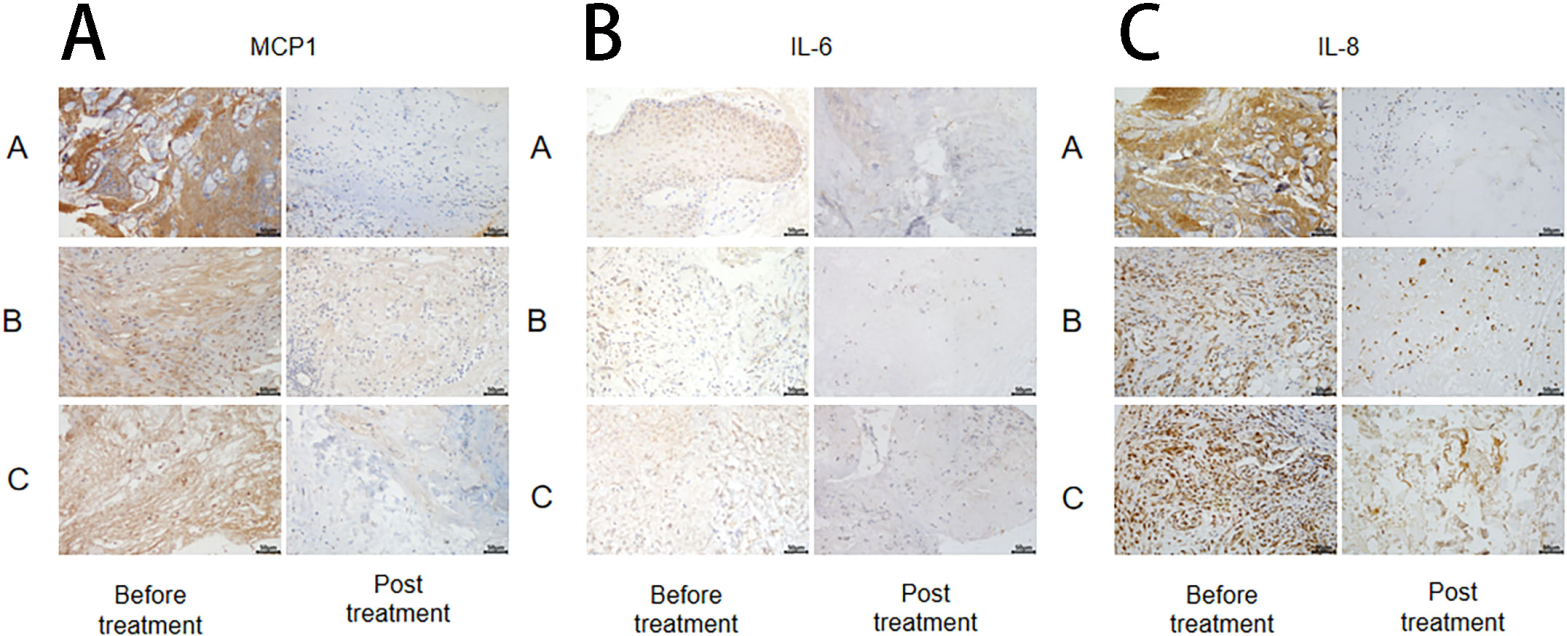
Immunohistochemical plots of MCP-1 **(A)**, IL-6 **(B)**, and IL-8 **(C)**.

The changes in inflammatory markers (MCP-1, IL-6, and IL-8) in wound tissue before and after treatment were detected using the immunohistochemical method in each group (**Figure 5**), followed by statistical analysis. The results revealed no significant difference in IL-6, IL-8, and MCP-1 levels among the groups before treatment (P > 0.05). (**Figure 6**).

## 4 Discussion

APG is a derivative of autologous PRP in gel form, functioning both as a carrier system for cell delivery and a release system for endogenous growth factors. Derived from blood extracted from the patient, APG minimizes the risk of rejection and immune responses associated with traditional PRP and has demonstrated efficacy in arthritis treatment (6). In recent years, attention has shifted toward its application in the treatment of DFUs.

The latest advancement in NPWT involves incorporating a flushing solution during the negative pressure process, thus combining NPWT with liquid perfusion. Commonly used irrigation solutions include physiological saline, antibiotics, insulin, silver nitrate, and select traditional Chinese medicines. This study presents a novel approach by using rh-aFGF as irrigation solution in negative pressure irrigation for the first time. The integration of the rh-aFGF+NPWT+APG system with conventional debridement and dressing yielded enhanced wound healing rates, reduced frequency of dressing changes, alleviated wound pain, decreased wound area, and shortened healing time and hospitalization duration. Mechanistically, this approach elevates SOD levels while reducing MDA to alleviate oxidative stress and suppresses the expression of inflammatory factors, resulting in significant clinical efficacy (7). However, findings related to angiogenic factors, particularly VEGF, are inconsistent in certain studies, reflecting controversies surrounding VEGF and its receptor in DFU research.

While many scholars suggest that down-regulation of VEGF is implicated in the pathogenesis of DFUs due to insufficient endothelial production resulting from vascular ischemia, conflicting evidence exists (8–10). Some studies indicate that elevated levels of VEGF facilitate wound healing, contradicting the findings of Drela et al. (11) Their study demonstrated a significant increase in pro-angiogenic factors among patients diagnosed with DFUS, with this elevation positively correlating with Wagner grade. As the DFUS syndrome improved, VEGF-A decreased compared to pre-treatment levels, indicating that heightened levels of pro-angiogenic factors in patients with DFS may be associated with lower limb ischemia and hypoxia. Furthermore, Mehmet et al. found no evidence indicating that VEGF and T-allele polymorphisms are risk factors for diabetes or DFU formation (12).

PEDF maintains a delicate equilibrium with VEGF *in vivo*. Functioning as an endogenous anti-VEGF factor, PEDF plays crucial physiological roles, including anti-inflammatory and antioxidant activities, inhibition of VEGF-induced angiogenesis and thrombosis, suppression of tumor formation, and neurotrophic effects (13, 14). Studies have indicated that the reduction in PEDF levels is negatively associated with the occurrence and progression of diabetic nephropathy, diabetic retinopathy, and the stability of coronary artery plaques, particularly in patients with chronic kidney disease facing an elevated risk of mortality.

Insufficient serum PEDF levels to counteract oxidative stress may contribute to increased vascular damage induced by oxidative stress, thereby elevating the risk of mortality in end-stage renal disease.

Both *in vitro* and *in vivo* investigations have demonstrated that exogenous supplementation of PEDF plays a pivotal role in combating VEGF-induced pathology and oxidative stress, while also regulating matrix metalloproteinases in conditions such as diabetic retinopathy and nephropathy. Consequently, PEDF holds potential therapeutic value for these complications (15, 16). Additionally, studies have indicated that PEDF exerts trophic effects on motor neurons while modulating non-neuronal recovery processes and enhancing neuroplasticity following ischemic spinal cord injury (17). In this study, an increase in PEDF and a decrease in VEGF were observed in the pathological tissue of patients with DFU during wound healing compared to pre-treatment levels. These seemingly contradictory results indicate that there may be distinct vascular reconstruction states within different tissue sites or stages of development. Various factors may regulate the activity of PEDF and VEGF in response to changing environmental conditions. We propose that the progression of DFU syndrome influences the concentrations of PEDF and VEGF, with particular emphasis on the pivotal role played by the PEDF/VEGF ratio *in vivo* in preventing and treating diabetic vascular diseases. Considering the significant physiological effects of PEDF, including its anti-inflammatory, anti-thrombotic, and antioxidant properties, the down-regulation of PEDF holds promise as a novel target for preventing and treating ischemic nerve injuries leading to DFU. However, further clinical and basic research exploration is required to ascertain whether supplementation of PEDF deficiency can enhance healing outcomes for ischemic neuropathic DFUs.

In this study, in addition to conventional treatment, exogenous growth factors were administered either as standalone interventions or in combination with NPWT and APG. The results indicated that the comprehensive treatment regimen involving APG in conjunction with NPWT and growth factors demonstrated superior clinical efficacy. This approach had a profound impact on ameliorating oxidative stress, reducing the local inflammatory response, and enhancing the equilibrium of VEGF to facilitate wound healing. Furthermore, it significantly reduced the frequency of dressing changes and alleviated wound pain while enhancing the cure rate of recalcitrant wounds. Therefore, this comprehensive treatment strategy holds substantial potential for practical application in clinical settings.

The results of this study suggest that the combination of APG with CVSD and rh-aFGF may become a systemic treatment for refractory DFU. The study included patients with Wagner grades 2-4 diabetic foot ulcers, covering a broad range. Implementing this treatment protocol in various clinical settings involves several practical aspects. Effective healing of diabetic foot ulcers relies on a comprehensive systemic treatment plan, which includes (1) Local wound debridement and dressing changes; (2) Use of appropriate antibiotics. (3) Management of the patient’s nutritional status and local blood circulation. (4) Application of NPWT to promote wound healing and reduce exudate, with high-dose rh-aFGF lavage administered before and during the negative pressure process to further enhance granulation tissue growth. (5) When suitable, for patients without contraindications to APG, selecting APG treatment based on the size of the wound and sinus tracts.

## 5 Conclusions

The comprehensive treatment involving APG combined with CVSD and rh-aFGF, in addition to conventional treatment, significantly enhances wound healing. It leads to a reduction in the frequency of dressing changes and wound pain, along with an enhancement in the cure rate of refractory wounds. This favorable outcome is likely attributable to a decrease in oxidative stress levels, modulation of the local inflammatory response, and an enhancement in the balance of VEGF. These findings indicate that after comprehensive treatment, patients experienced higher ulcer healing rates and faster healing times. During the follow-up period, patients had a lower rate of ulcer recurrence and improved quality of life. However, the recurrence of diabetic foot ulcers may be influenced by various factors, including the level of diabetes control, vascular conditions, overall patient health, and adherence to treatment. It is generally recommended that patients undergo regular clinical evaluations and imaging studies to monitor ulcer healing and detect potential complications. These follow-ups are crucial for timely adjustments to the treatment plan and to ensure optimal treatment outcomes. While PRP treatment for diabetic foot ulcers has shown promising long-term effects, further randomized controlled trials and long-term follow-up studies are needed to validate its efficacy and safety.

## Data Availability

The original contributions presented in the study are included in the article/supplementary material. Further inquiries can be directed to the corresponding author.
